# The Australian mental health system: An economic overview and some research issues

**DOI:** 10.1186/1752-4458-2-4

**Published:** 2008-05-14

**Authors:** Ruth FG Williams, DP Doessel

**Affiliations:** 1 School of Applied Economics, and Centre for Strategic Economic Studies, Victoria University, Melbourne, Australia; 2 Queensland Centre for Mental Health Research, "The Park" – Centre for Mental Health, Australia; 3 School of Population Health, The University of Queensland, Brisbane, Australia

## Abstract

This article is concerned with the *key economic characteristics *of Australia's mental health system. First, some brief conceptual and empirical descriptions are provided of Australia's mental health services, both as a total system, and of its two principal components, *viz. *public psychiatric institutions and private psychiatry services. Expenditures on public psychiatric hospitals clearly demonstrate the effect of deinstitutionalisation. Data from 1984 on private practice psychiatry indicate that per capita utilisation rates peaked in 1996 and have since fallen. Generally, since 1984 gross fees have not risen. However, for both utilisation and fees, there is evidence (of a statistical kind) that there are significant differences between the states of Australia, in these two variables (utilisation and fees). Emphasis is also placed on the economic incentives that arise from health insurance and the heterogeneous nature of mental illness. The effects of these incentives are regarded as by-products of the health insurance mechanism; and another effect, "unmet need" and "met non-need", is a somewhat unique problem of an informational kind. Discussion of many of these issues concludes on a somewhat negative note, e.g. that no empirical results are available to quantify the particular effect that is discussed. This is a manifestation of the *lacunae *of economic studies of the mental health sector.

## Introduction

Mental illnesses and conditions are amongst the most important chronic diseases in populations internationally [1], and in Australia [2-4]. Mental health problems are receiving more attention, and mental illnesses are now less stigmatised, in the general population and in several scholarly literatures, than they have been for centuries. Topics in the media and the literatures of mental health professionals and health services researchers indicate broad concerns over homelessness, youth suicide, the prevalence of depression, the drugs of dependence and the side-effects of medications, and so forth. However, some other mental health issues receive relatively less attention, and some disciplines, including economics, tend to neglect mental health issues.

The purpose of this article is to *draw attention to the major economic features *of Australia's mental health system or sector. The initial Section emphasises the heterogeneity of the sector using a Venn diagram that depicts the main sets, i.e. the components of the system. The Section also presents some data on how the size of public psychiatric institutions has changed through time, as well as providing an overview of the key economic features of private psychiatry services. Emphasis is then placed on the incentives or motivations of participants in the health system: this discussion is centred on issues associated with health insurance *per se*, *viz. *moral hazard and two issues of an informational kind (adverse selection and an imperfect agency relationship), as well as the twin issues of "unmet need" and "met non-need". Another Section considers the importance of the range in the severity of diagnoses, as well as heterogeneity in general, and the implications for mental health services. There is a brief conclusion that highlights the paucity of economic studies of mental health in Australia.

## An economic overview of Australia's mental health system

Heterogeneity characterises Australia's mental health system: multiplicities of services are provided, and there are multiplicities of localities where these services are provided. No "norm" ought necessarily be concluded from casual observation.

Another characteristic is that the boundaries of the segments are somewhat arbitrary when one attempts to comprehend the system. A "narrow", "traditional" or "conventional" focus on the system is available by applying National Health Accounting conceptions. These conceptions were defined by Abel-Smith [5], the architect of the World Health Organization (WHO) classification of health expenditure. Broader conceptions, involving all relevant economic phenomena, can also be defined. These are discussed shortly.

**Figure 1:**
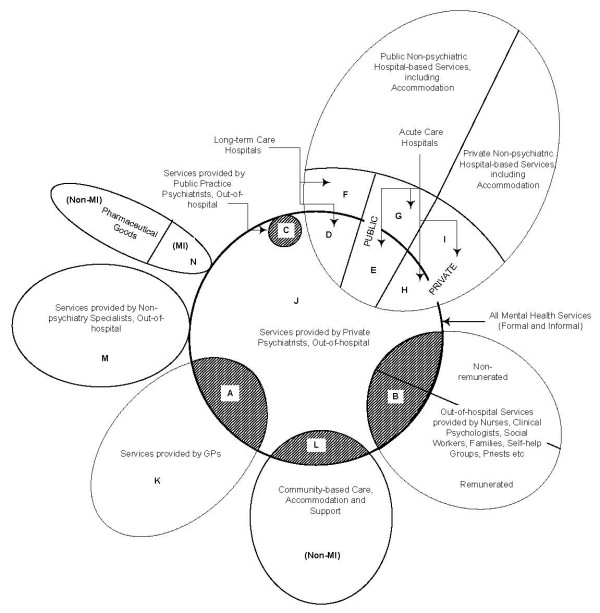
A Schematic Conception of the Jigsaw of "Conventional" Mental Health Services.

The multiplicity of service providers is depicted conceptually as a Venn diagram in Figure 1[6]. This Figure is constructed with "All Mental Health Services (Formal and Informal)" at the centre. Each of the other sets represents other parts of the health sector, some of which do not relate to mental health at all, for example "Private Non-psychiatric Hospital-based Services, including Accommodation", and "Non-Mental Illness (MI) Pharmaceuticals". The universal set is the health sector. Individual sets depict a service provider, e.g. GPs, specialist psychiatrists, para-medical professionals, family members, self-help groups and so on. Some sets are remunerated for their service provision and others are not.

The Figure indicates that services are provided in a number of locations. Although location can largely be simplified to "in-hospital" and "out-of-hospital", there is an important distinction to be made between dedicated public psychiatric hospitals that provide long-term care for the chronically ill, and short-term hospital services for the acutely ill. (Chronic/acute mental illnesses are not always diagnostically clear and mental illness can, for some diagnoses, be conceived on a continuum. This issue will be discussed below in relation to Figure 3, which involves a consideration of heterogeneity). Note also that the provision of long-term care has decreased since the advent of the deinstitutionalisation movement, which resulted in community-based service provision. Deinstitutionalisation is not a recent phenomenon: Australia's experience began in the mid-1950s [7]. Figure 1 also shows that mental health services can also be provided in dedicated private psychiatric hospitals.

Although Figure 1 presents the main elements of the system, it does not capture all the "messiness" that arises from the dynamics underlying the health sector. Changes in funding/insurance arrangements, the temporal ebb-and-flow of federal structures and so forth are not depicted.

Despite Figure 1 being schematic in nature, it can be used to indicate empirical measures of various concepts. Suppose one sought to determine the level of expenditure on all mental health services (except forunremunerated services and opportunity costs, as well as intangible costs such as pain or suffering.) Figure 1 indicates that this exercise would involve the summation of expenditures associated with the various areas, depending on how comprehensive is the conception of "mental health expenditure". The nine areas A + B + C + D + E + H + J + L + N would provide a narrow conception. If one has a slightly wider conception, that is, all mental health treatments/services and hospital accommodation, the focus would be on the following twelve segments in Figure 1: A + B + C + D + E + F + G + H + I + J + L + N. However, a comprehensive economic conception of mental health expenditure, conceived of in the Cost-of-Illness (COI) approach, would include these twelve areas, as well as opportunity costs (lost earnings) and intangible costs (disability, pain, suffering, loss etc.), which are not specifically indicated in Figure 1. Examples of such COI studies are those for schizophrenia [8] and bipolar disorder [9]. Thus, Figure 1 serves as a guide to the mental health system and, in addition, indicates the relevant variables, when matters of measurement and quantification are the subject of attention.

One partial expenditure estimate of the size of the "system" is available for 2000–01 from the Australian Institute of Health and Welfare (AIHW), as part of an estimation exercise of ("direct") or "accounting" health expenditures by disease groups [10,11]. Mental illness (excluding community mental health services) accounted for $3,018 m of $49,174 m total "allocated" health expenditures in that year. This estimate is smaller than a comprehensive estimate based on an (economic) COI conception discussed above. However, in terms of this narrow estimate, mental illness is the seventh most expensive disease category in Australia.

### Public psychiatric institutions

Some numerical time-series data on patients in public mental hospitals from 1906 are available for Australia [12]. Some States also have more detailed data sets, e.g. Queensland from 1883–84. However, time-series economic data are not available before 1960–61, when Deeble [13,14] collected data on a classification "Public Psychiatric Hospitals", a component of the WHO expenditure classification system. Subsequently, the Australian Institute of Health and Welfare (AIHW) has routinely collected such annual data.

**Figure 2:**
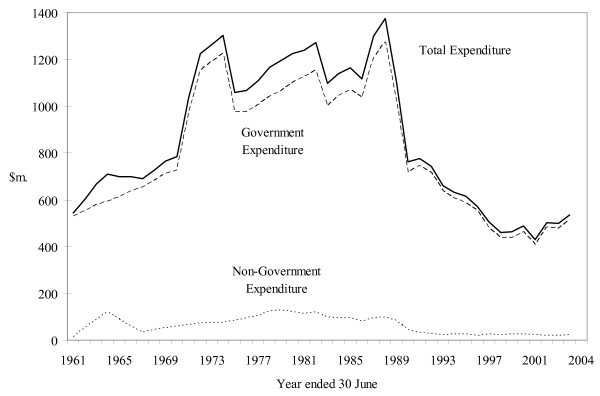
**Government, Non-Government and Total Expenditure on Public Psychiatric Hospitals, 1960–61 to 2003–04, $m. (2003–04 Prices).Note: **Australian data for the following years have been estimated by linear interpolation: 1961–62, 1962–63, 1964–65, 1965–66, 1967–68, 1968–69 and 1970–71.

Figure 2 presents the available expenditure data for Public Psychiatric Hospitals from 1960–61 by Government and Non-government source of funds, in constant (2003–04) prices. Note that non-government funds are clearly trivial (in a quantitative sense): chronic mental illness is regarded as the responsibility of government. The overall inverted, U-shaped trend is explained, in large part, by the deinstitutionalisation process in Australia.

Figure 2 incorporates grouped data for six separate state-based systems. It should be noted that the aggregated nature of this Figure conceals differences between the states in terms of scope and data coverage.

### Private psychiatry services as an industry

The psychiatry services industry in Australia involves psychiatrists in private and public practice who work in the mental health sector. Although medical practitioners in "general practice" (GPs) also provide some services of a psychiatry kind, private psychiatry is defined (as an industry) by post-graduate education in psychiatry theory and practice, and examined by a specialist College. When the required level of education is attained, a medical practitioner may be admitted to College membership (the "guild"). "Guild" is an historical term, but is used quite deliberately by some authors in regard to the health sector, e.g. Enthoven [15]. In Australia, the relevant "guild" is the Royal Australian and New Zealand College of Psychiatrists (RANZCP). In 2004 there were 2,409 fully qualified psychiatrists in Australia, and 742 medical practitioners undertaking some training to become psychiatrists [16].

In terms of producing services that require prescriptions for pharmaceuticals, the entry requirements into the industry involve the aforementioned medical qualifications (from a university) and the required periods of supervised practice, as well as registration with a Medical Board. Then, for consumers to claim a subsidy under Australia's health funding arrangements (Medicare), a psychiatrist requires a Provider Number issued by Medicare Australia. These requirements apply in both public and private medical practice, except that in the public sector medical practitioners do not need a Provider Number.

From an economic perspective, membership of the RANZCP is a key characteristic of entry into the industry. It is a necessary condition for charging the specialist fees associated with the psychiatry services, subsidised by Medicare, which are listed and defined in Section 8 of the *Medicare Benefits Schedule*[17]. Due to the definitions in the *Schedule*, private psychiatry services can be regarded as well-defined products.

### Economic Incentives under (Medicare) Insurance

In Australia, private medical practitioners operate under a universal, compulsory health insurance scheme, Medicare, which is financed from Australian Government general taxation revenue and an earmarked tax (the Medicare levy). In effect, Medicare subsidises all private fee-for-service (FFS) medical consultations, including private psychiatry services. There are complex relationships between gross prices, net ("out-of-pocket") prices, the Medicare rebates (subsidies) and Schedule Fees. The relationships between these four variables are considered in detail elsewhere [18].

The *raison d'être *for Medicare (and its 1975 predecessor, Medibank) is the enabling of "equal access" for Australians to basic medical, hospital (and optical) services. Its purposes are asserted to be: " [m]ore equitable financing arrangements, a redistribution of income to the poorer members of the community, the extension of benefits to people not previously covered, a greater measure of influence over the fees charged by doctors, more efficient administrative procedures and an improved information system for the monitoring of medical service provision" [19].

We now turn to a discussion of some economic side effects of health insurance. This discussion is directed to two specific issues, *viz*. incentives created by insurance *per se*, and secondly the particular context of (Australia's) Medicare.

There is a long-standing literature in economics, stemming from Arrow's seminal paper [20], that health insurance mechanisms (whether public or private) involve various incentives for consumers, producers and insurance carriers. Arrow's argument was that the health sector is characterised by various non-market institutional arrangements due to the inability of markets to deal with some of the unique features of health. However, one of the characteristics of heath and illness, *viz*. the uncertainty of illness (particularly catastrophic expenses associated with some illness), leads to insurance. This is a commonly applied mechanism to handle uncertainty in economic affairs, whether the uncertainty arises in the events of car accidents, illness, death, theft, fire etc. Although insurance improves people's welfare (via the pooling of risks and thus alleviating catastrophic losses), other problems are associated with insurance *per se*. The two major problems are referred to as moral hazard and adverse selection; and both of these problems are associated with people's incentives.

Moral hazard refers to the situation that arises when, because of insurance coverage, the out-of-pocket price of health services falls and, as a result of this price fall, consumers purchase more health services than they would have done in the absence of health insurance (assuming that the demand for these health services is not perfectly inelastic). Whether or not this behaviour is "moral" can be debated elsewhere; what is of significance here is that this behaviour is the predictable response of a consumer to a price fall, and that a higher quantity demanded results when a particular set of incentives are operational under health insurance coverage. This behaviour of increasing consumption when price falls, when summed across a population, leads to non-trivial welfare effects in the long run. This phenomenon can be likened to the case where a small act of, say, littering or pollution by a single individual has a trivial effect, but the sum total of widespread small acts of littering or pollution by all individuals is not trivial: these acts impose major impacts that a community bears jointly. For some of the contrasting moral issues, see the perspectives of Pauly [21] and, in another context, Kahn [22], the author of the expression,"the tyranny of small decisions". However, from the stance of the economic effects, the increased demand gives rise to a welfare loss (of a general, or population-wide, nature) that comes from the breaking of the nexus between the costs of services and the prices charged for them. This welfare loss negatively affects (to some extent) the general welfare gain from the pooling of risks.

There are no empirical studies of the welfare cost associated with moral hazard in mental health services in Australia, and one of the key variables, the value of the own-price elasticity of demand for mental health services, is also not known in Australia. Thus, quantification of these phenomena is an item outstanding on the research agenda.

The second problem mentioned above, adverse selection, arises from the fact that health insurance attracts people who are likely to be large or "heavy" consumers of health services, generally people with low health status, whereas people with high health status are not inclined to buy such insurance. Once more, there is an incentive effect. This issue arises since consumers possess more information about their health status and their personal demand for health services than do insurance carriers. This is true for both "the ill" and "the healthy". Thus, we have a case of asymmetric information, having the effect that the people with higher health risks tend to drive the low risks from the health insurance market. If the low risks stay in the market, there will then be a redistribution of income (in terms of the difference between the amount of premium that a member pays and the benefits received) from the low risks to the high risks.

Under the compulsory membership arrangements of Medicare, adverse selection does not exist, but it does exist under private health insurance arrangements in Australia. Nobody in Australia discusses, let alone investigates, adverse selection with respect to the use of mental health services. Quantification of this phenomenon is another outstanding item for an economic research agenda about Australia's mental health system.

There is yet another informational problem that arises in the health sector, which is appropriately described as one of imperfect information. Generally, consumers possess less information than medical professionals about diagnosis and appropriate therapy. The presence of information differentials over the daily problems of living explains why consumers consult professionals of various kinds (lawyers, accountants etc.), as well as health professionals. The common characteristic of all such relationships is that of agency, i.e. where a "principal" (such as a patient) employs an agent (a medical practitioner) to act on his/her behalf, for a fee. These conceptions quickly lead to consideration of whether the agency relationship is perfect or imperfect. A perfect relationship is one in which the agent aligns his/her decision-making with the principal's (consumer's) preferences: where there is a conflict between the producer's interest and the consumer's interests, the perfect agent will implement actions in line with the consumer's (i.e. agent's) preferences. An imperfect relationship, on the other hand, arises when the producer (agent) makes decisions for the principal (consumer) that serve the interests of the producer. These issues are discussed in various texts, such as Folland, Goodman and Stano [23] and Phelps [24].

This imperfect agency relationship lies at the heart of the supplier-induced demand hypothesis, introduced to the health insurance literature by Fuchs and Kramer [25] and Evans [26]. See also Fuchs [27]. Essentially, this hypothesis is tested by inserting a supply variable (e.g. the number of medical practitioners) in the estimation of a demand equation for medical services. Work (on GPs and specialists in aggregate) in this *genre *has been undertaken on Australian data by Richardson [28].

Supplier-induced demand has been one of the most contentious areas of research in health economics. Space precludes a survey of this extensive literature, but it is useful to note that replication of the major studies that brought this hypothesis on to research agendae has cast serious doubt on it. Consider this statement by Ramsay and Wasow [29]:

The empirical findings of the early researchers on the issue of supplier induced demand are most likely the result of improperly specified and inadequately analysed regression models. No, or certainly very few, useful empirical generalisations can be drawn from any of the models as currently formulated and estimated. In this sense, the anomalous empirical findings are statistical artefacts (p. 67).

It must be noted that, just as there is no empirical evidence on moral hazard and asymmetric information, with respect to Australia's mental health sector, there is no empirical evidence on this third issue, just discussed.

However, there are some studies that can be interpreted as shedding some light into these various "dark corners" of the economics of the mental health sector. In 1996, the Australian Government placed financial constraints on the number of consultations that specialist psychiatrists could have with an individual patient in a calendar year. In one case, reported in the House of Representatives by the (then) Minister for Health, a psychiatrist had 747 individual consultations with a single patient in a particular year. Essentially, the changes introduced at that time restricted consumers to 50 such consultations per year, under standard Medicare subsidies, and any consultations in excess of 50 entailed a quite different subsidy/rebate arrangement. An evaluation of the effectiveness of these arrangements is now available [30].

It could be argued that the background to these changes to the *Medicare Benefits Schedule *in 1996 involved the self-interest of these psychiatrists who were "over-servicing" a small number of patients under Australia's FFS system of remuneration of private practice medicine. Such a view is too simplistic. Some patients do require, at certain times, intensive consultations with mental health professionals, a point recognised by the Australian Government with the introduction of Item 319 into the *Medicare Benefits Schedule*. In addition, numerous consultations with psychiatrists may be patient-initiated and motivated by the patient's preferences. A third possibility is that such numerous consultations may be a manifestation of a symbiotic consumer-producer relationship. However there is no empirical study which has "unscrambled" this "omelette", and hence any comment amounts to speculation.

There are some epidemiological data (from the 1997 national survey, *Mental Health and Wellbeing...*[31]) which indicate that there is some cause for concern as to the operational efficacy of the nation's mental health system. This ABS study is based on a representative sample survey of Australian adults, and generated data, not only on the prevalence of mental illness, but also the use of mental health services by people with mental illness, and people *without *mental illness. This has led to some analysis using concepts of "unmet need" and "met non-need" etc. Such work by Andrews [32] indicates that the problem of "unmet need" is far from trivial. See also Whiteford [33]. Further work on this issue with an equal emphasis on "met non-need" is currently being undertaken [34]. The incentives, or the preferences, that lie behind this phenomenon of mental health services being provided to people without mental illness, is a fertile field for further work.

### Economic outcomes

There is a dearth of Australian studies that engage in descriptive economic science about the impact of Medicare in the mental health sector. We now turn to some economic outcomes of Australia's private FFS psychiatry services under Medicare.

In this section two variables of interest are examined using quarterly economic data, supplied by the (then) Commonwealth Department of Health and Ageing (CDHA) for the period between the start of Medicare in 1984 and 2001 [35]. The two variables are the quantities of all private FFS psychiatry services produced and consumed, and the gross prices (i.e. inclusive of the Medicare subsidy/rebate) for these services. The quantities of services are aggregated across the relevant Item Numbers in the *Medicare Benefits Schedule *[36]. While other variables in the industry are also relevant, e.g. net prices, analysis of such other variables is not possible in this paper.

To provide a general context, Table 1 indicates that 32,757,227 psychiatry services were funded under Medicare between 1984(1) and 2001(3). Table 1 also shows a disaggregation by sole consultation (and temporal length), group consultation, ECT services and interviews. Sole consultations dominate (95.11 per cent of all services), and the 46–75 minute consultation represents approximately one-half (or 47.0 per cent) of total services.

**Table 1:** Total Numbers (and Percentages) of Psychiatric Services Aggregated in an Eightfold Classification of Medicare Items, 1984(1) To 2001(3), Australia.

Service Category by Groups of Items	No. of Services	Percentage	Service Category by Groups of Items	No. of Services	Percentage
1–15 mins duration^(i)^	1,884,004	5.75	Group Psychotherapy^(vi)^	1,341,216	4.09
16–30 mins duration^(ii)^	6,115,787	18.67	Other than Patient^(vii)^	76,177	0.23
31–45 mins duration^(iii)^	6,802,651	20.77	E.C.T.^(viii)^	180,027	0.55
46–75 mins duration^(iv)^	15,401,695	47.02	Suppressed Details^(ix)^	6,994	0.02
> 75 mins duration^(v)^	948,676	2.90	Total Services	**32,757,227**	**100.00**

**Notes: **(i) Items 134, 300, 310, 320 and 330

(ii) Items 138, 304, 314, 324 and 334

(iii) Items 136, 302, 312, 322 and 332

(iv) Items 140, 306, 316, 326 and 336

(v) Items 142, 308, 318, 328 and 338

(vi)Items 342, 344 and 346

(vi) Items 348, 350 and 352

(viii) Item 14224

(ix) "Suppressed Details" refer to the number of services subject to confidentiality restrictions on data supplied by the CDHA.

Table 2 presents summary statistics on quantities of total FFS psychiatry services per 1,000 population for six regions [37]. Note that each of the Territories is aggregated with a State. These aggregations, undertaken by the (then) CDHA, are a consequence of the confidentiality conditions surrounding the occurrence of "small cells" generated by some Items. Hence, the regions are as follows: NSW is aggregated with the ACT (NSW/ACT); Victoria (Vic.); Queensland (Qld); South Australia is aggregated with the Northern Territory (SA/NT); Western Australia (WA); and Tasmania (Tas.). And there is a seventh, i.e. the total for Australia. Table 2 suggests spatial variation at a regional level in psychiatry utilisation rates.

**Table 2:** Summary Statistics on Private Psychiatric Services per 1,000 Persons, States/Territories, Australia, 1984(3) to 2001(3)

	NSW/ACT	Victoria	Queensland	SA/NT	WA	Tasmania
Mean	26.4	33.8	24.7	28.4	15.1	19.8
S.D.	2.8	5.6	2.9	4.8	2.5	4.0
Range	20.4 to 32.0	21.1 to 42.5	18.2 to 30.1	18.7 to 36.6	10.3 to 21.6	12.3 to 25.9

**Notes: **(i) Items 134, 300, 310, 320 and 330

(ii) Items 138, 304, 314, 324 and 334

(iii) Items 136, 302, 312, 322 and 332

(iv) Items 140, 306, 316, 326 and 336

(vii) Items 142, 308, 318, 328 and 338

(vi) Items 342, 344 and 346

(viii) Items 348, 350 and 352

(viii) Item 14224

(x) "Suppressed Details" refer to the number of services subject to confidentiality restrictions on data supplied by the CDHA.

**Figure 3:**
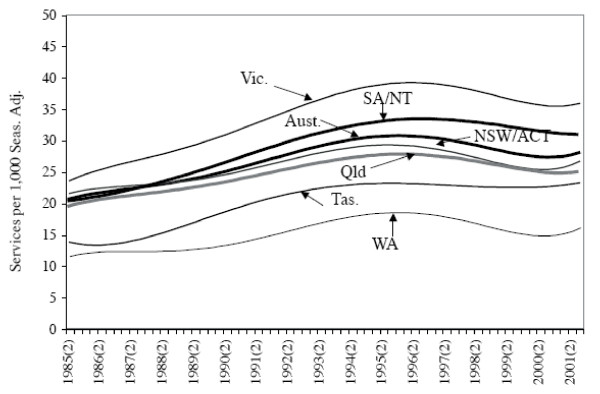
**Estimated Intercept Coefficients for Estimated Equations on Private Psychiatric Services per 1,000 Persons, States/Territories and Australia, 1984(3) to 2001(3)**. **Notes:** (i) The notation 1984(3) refers to the third (September) quarter of 1984, etc. (ii) The psychiatric services referred to here relate to the aggregate of the Item Numbers listed in the Notes to Table 1.

The data summarised in Table 2 have also been examined statistically [38]. This study is concerned to determine statistically whether the temporal utilisation rate, for each region, has increased, decreased, or remained constant under Medicare. Quarterly intercept coefficients are derived from equations estimated for each region on private psychiatry services per 1,000 persons from 1984(3) to 2001(3). See Figure 3[36,37]. Note that the per capita utilisation rate for Australia (and all regions, except Tasmania) reached a maximum in 1996, and has since declined. Statistical analysis of the equations using Wald coefficient restriction tests of the coefficients is also undertaken in order to test for equality of (spatial) access. This reveals statistically significant differences between the regions in estimated coefficient values. Thus, it can be concluded that utilisation rates for private psychiatry services are not spatially and temporally uniform in Australia under Medicare.

The utilisation rates, just reported, for private psychiatry services across the regions of Australia during the Medicare period need to be considered in the light of epidemiological information. The studies of Burgess, Pirkis, Buckingham *et al. *[39], Blazer, George, Landerman *et al. *[40], and Kendler, Gallagher, Abelson and Kessler [41] all demonstrate clearly that the prevalence of mental illnesses across some populations is relatively uniform. Thus, the likely explanation of the Williams and Doessel result on different utilisation rates lies in economic factors, i.e. "access". Access involves demand variables (such as price, income), supply variables (such as the number and distribution of psychiatrists), various socio-economic characteristics of the relevant populations, and remoteness. Clearly, further study is needed of the economic factors determining the outcomes of the market/s for private psychiatry services under Medicare.

It is also relevant to ask what is known about gross prices for private psychiatry services at the regional level. Summary statistics are reported in Table 3 on quarterly data (expressed in 1989–90 prices) [35,37]. These summary statistics show that, in four regions, NSW/ACT, Vic., SA/NT and WA, the average gross price is similar, approximately $84 to $85. The maximum average gross price occurred in NSW/ACT. The standard deviations in those regions are also similar. Note that the average gross price is considerably less in the other two regions, Tas. and Qld. The standard deviation for Qld is the lowest, whilst WA has the highest standard deviation. The ranges presented in Table 3 also indicate large temporal variations from one region to the next.

**Table 3:** Summary Statistics on Gross Prices for Private Psychiatric Services for the States/Territories, Australia, $s (1989–90 Prices), 1984(3) to 2001(3)

	NSW/ACT	Victoria	Queensland	SA/NT	WA	Tasmania
Mean	85.38	84.02	77.63	83.95	84.95	75.12
S.D.	2.95	2.09	1.66	3.17	3.05	4.29
Range	81.11 to 94.60	77.44 to 87.12	74.30 to 81.11	78.05 to 90.02	77.34 to 90.36	64.58 to 83.26

**Notes: **(i) Items 134, 300, 310, 320 and 330

(ii) Items 138, 304, 314, 324 and 334

(iii) Items 136, 302, 312, 322 and 332

(iv) Items 140, 306, 316, 326 and 336

(ix) Items 142, 308, 318, 328 and 338

(vi) Items 342, 344 and 346

(x) Items 348, 350 and 352

(viii) Item 14224

(xi) "Suppressed Details" refer to the number of services subject to confidentiality restrictions on data supplied by the CDHA.

**Figure 4:**
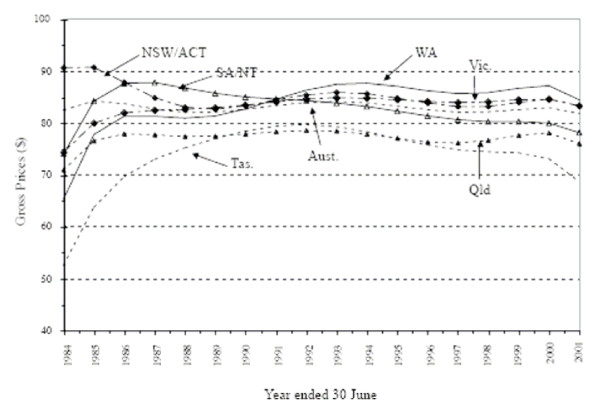
**Estimated Intercept Coefficients for Estimated Equations on Gross Prices for Private Psychiatric Services, States/Territories, Australia, $s (1989–90 Prices), 1984(3) to 2001(3)**. **Note: **Australian data for the following years have been estimated by linear interpolation: 1961–62, 1962–63, 1964–65, 1965–66, 1967–68, 1968–69 and 1970–71.

These quarterly gross price data since 1984 for the six regions of Australia have also been analysed statistically [42]. Figure 4 shows quarterly intercept coefficients for equations on the gross price data, estimated for each Australian region from 1984(3) to 2001(3). One overall result from this study is that gross prices (in 1989–90 prices) for the services of private psychiatrists are found to have remained fairly constant during the study period for all six regions. This is an interesting outcome, since the general perception is probably that these prices have risen over time. However, note that this overall conclusion hides a "mixed picture" in some regions: prices fell in SA/NT between 1984 and 2001, rose in WA during that period, and fell in Tas., although the magnitudes of these exceptions are not large. It ought to be noted that whether the productivity of private psychiatry treatment in this period of time has risen, fallen or remained constant, is not known for Australia. Further analysis of the differences in the equations, using Wald coefficient restriction tests, was undertaken. This analysis shows that, although spatial price differences for private psychiatry services predominate, there is, in all, a mixed picture, spatially, of price similarities and differences.

It should be noted that economic issues can also be studied in the sub-sets of psychiatry Items in the Medicare Schedule. For example, a single economic study is available for private sector ECT utilisation rates across the regions of Australia [43].

Much additional analysis is required of Medicare data from a range of economic perspectives. Apart from developing explanatory models using regression analysis to determine the factors that explain utilisation patterns, such as those already described, the analysis of the data at a lower level of geographical aggregation than the broad state/territory regions of Australia is important. Doing so will measure the likely extent of differences in utilisation between metropolitan, minor city, rural and remote regions of Australia. The same applies to gross and net prices. Note also that the construction of price indices for psychiatry services is possible also from Medicare data. Moreover, the application of the conventional measures of economic inequality and concentration employed by economists to mental health variables is relevant.

The available economic studies on Australian mental health issues are extensively reviewed in Williams and Doessel [44,45]. The earlier of these two surveys [44] provides the more comprehensive account of the state of empirical economic knowledge, at the time of writing, about the mental health system. The survey includes not only economic studies but also the important contributions from (mental) health services research. Also included in the survey are the studies about the "grey areas" of the mental health system, i.e. economic research about the two main co-morbidities, intellectual disability and substance abuse. The studies that have been undertaken by psychiatrists, psychologists and other health professionals in Australia, who have crossed their own discipline boundaries in order to address the pressing issues of the mental health system, are also highlighted.

Having described some economic dimensions of Australia's mental health system, the focus will turn to a brief discussion of the relationship between mental illness and the mental health sector.

## Australia's mental health system and mental illness

Mental disorders involve a broad spectrum of illnesses and conditions whose impact ranges from being extremely disabling to quite mild [46,47]. As pointed out above [32], only a fraction of people with mental illness consumes mental health services: thus many, even with psychoses, have no contact with the mental health system. On the other hand there are many people (who do not meet the criteria of a mental illness) who do consume mental health services. The motive for this utilisation may be for the purposes of achievement or self-fulfilment [48,49]. Others, the "worried well" [50,51], also seek help, but their conditions are not within the conventional diagnostic criteria of mental illness. There are no Australian studies that indicate the relative importance of the effects of these various motives.

**Figure 5:**
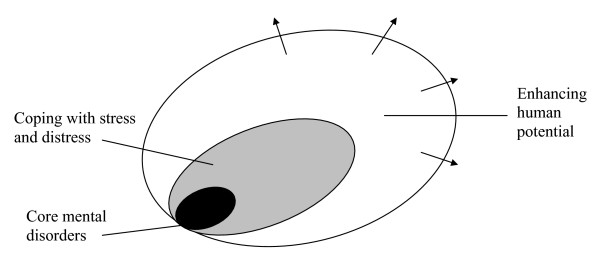
The Diagnostic Spectrum.

A stylisation of the groups described in the previous paragraph is provided in Figure 5[52], which presents a distinction between serious mental illness at the core and mild conditions that are commonly experienced difficulties in coping with stress and distress, and thirdly the reasons for seeking help that involve those who wish to enhance themselves in their life roles. The distinction between these groups is not a trivial concern, as the funding of health services enables the non-sick to access government-subsidised or government-provided services. Given the private nature (in the economic sense) of medical services, such behaviour denies these scarce services to the mentally ill.

It is often suggested that allocated resources to Australia's mental health system are inadequate. However, the concern that Andrews [32] and Whiteford [33] express is the dilemma of "structural imbalance". Doessel, Williams and Nolan [34] demonstrate the dimensions of this phenomenon, and argue that "throwing more money" at the pre-existing structures in Australia will have little effect on the structural imbalance in Australia's mental health system. They suggest some possible measures for reform to address this structural imbalance dilemma.

## Conclusion

This paper has addressed a relatively small number of questions associated with Australia's mental health sector, and the approach taken is, in large part, from the discipline of economics. For example, the emphasis placed on incentives is well established as an approach relevant to the scope of economics. This is not to suggest that other approaches, say, that of a health services research kind, are not relevant. It has been argued elsewhere that these are complementary (not substitutable) ways of analysing mental health issues [53]. These two approaches ask different, but related, questions.

Mental health clearly is a relatively untilled field for economists. Many empirical gaps exist in regard to both the private and public sectors. Also, work at a more conceptual level is very relevant to publicly provided mental health services, such as LeGrand's analyses with respect to the types of structural changes appropriate to the (non-market) National Health Service (NHS) in Britain [54-57].

Researchers of the economics of Australia's mental health system are likely to find no shortage of topics, but conceptual and practical problems are more intractable in the analysis of mental health than in health care generally. The crafting of research questions is of paramount importance. Also, some of the characteristics of mental health services have more in common with markets for disability services than medical services.

## Competing interests

The authors declare that they have no competing interests.

## Authors' contributions

RFGW and DPD contributed jointly to the conception, design, coordination, participation and the writing process. RFGW and DPD read and approved of the final version.
